# Supplementary material for: Government subsidies and innovation in new energy vehicle companies: An empirical study of new energy vehicle listed companies based on Shanghai and Shenzhen A-shares
Source: PLoS One. 2023 Apr 20;18(4):e0284693. doi: 10.1371/journal.pone.0284693 (PMC10118184; doi:10.1371/journal.pone.0284693)
Supplement: Attachment — Submitted filename: Review_Report_PONE-D-22-30783.docx [file pone.0284693.s002.docx]

Reviewer’s Report on Manuscript PONE-D-22-30783 titled “Government subsidies and innovation in new energy vehicle companies: Empirical study of new energy vehicle listed companies based on Shanghai and Shenzhen A-shares”

This paper studies the effect of government subsidies on corporate innovation of new energy vehicle enterprises (NEV) in China using panel regression model. The results show that the effects of government subsidies on NEV innovation is non-linear and heterogeneous depending on the role of firms in the value chain of industry, the firm age and type of firms. Particularly, the innovation effect is significant for downstream vehicle enterprises, but no such effect for midstream core component and upstream raw material enterprises. Further, the innovation effect of subsidies is more pronounced in young enterprises compared to old enterprises. In addition, the subsidies only induce significant effects on innovation of non-state-owned firms while the innovations of state-owned firms are not affected by government subsidies.

The NEV has recently emerged as the strategic industry in China’s Industry Development Plan to achieve the country’s low-carbon emission and sustainable energy consumption targets, thereby the literature on the determinants of corporate innovation in China NEV industry has gained extensive attention from both researchers and practitioners. To my knowledge, there are abundant studies investigating the innovation effect of government subsidies on NEV firms, including Jiang et al. (2018), Li et al. (2021), Shao et al. (2021), to name a few. These papers all reveal that there is non-linear relationship between government subsidies and innovation in NEV firms in China. Further, the innovation effect intensity is region-dependent, varies by the type of subsidies and the type of enterprises.

This paper aims to extend the literature by providing the evidence of an inverted U-shaped relationship between government subsidies on NEV firm innovation in China. However, in the introduction section, the authors only state the research subject which is investigating the effect of government subsidies on firm innovation. Thereby, they failed to differentiate their study from the existing studies in term of research issue. That is, the paper limits its academic contribution. Further, the other main contribution of the paper is investigating how the effect of government subsidies on firm innovation changes depending on the role of enterprises in value chain of the industry, the type of firms (state-owned firms vs non-state-owned firms) and the firm age. So, in the current introduction section, the authors should provide appropriate arguments and explanations to highlight this main contribution of the paper. The study also needs more in-depth analyses regarding methodology, variable selection and empirical analyses.

So, I have several comments/ suggestions to improve the quality as well as the contribution of the paper, as follows:

1. The current introduction seems to provide too much background information regarding China NEV industry while the motivation and contribution are not made clear. I suggest the following:

- Shorten the background information, brief review of the relation between government subsidies and firm innovation in general (i.e., existing evidence of non-linear relationship, crowding-out effect in this relationship),
- State the research subject of the paper in the first/ second paragraph of the introduction which is different with the existing studies as it first provides the evidence of inverted U-shape relationship on NEV firm innovation in China.
- State the reason why it is important to explore how the effect of government subsidies varies by the role of firms in value chain, the firm type, and the firm age. This would help highlight the main motivation and contribution of the paper in introduction section.

2. In literature review, the paper provides appropriate hypothesis developments. However, regarding the hypothesis 1, I suggest citing more relevant and recent literature as mentioned above. Please check the duplicate sentences (which happen quite frequently) and redraft the literature review section.
3. Regarding variable selection, I perceive that there are many wrong descriptions of the variables used in the model. For example, “Patent­­_it_ is the explanatory variable”??? More importantly, the authors did not provide the reasons why they only use Patent as a proxy for firm innovation. Since the major existing study documents that firm innovation is an input-output process which could be proxied by R&D spending and the number of the patents. (Li et al., 2021). On this matter, I would prefer to see more detailed explanation on the selection of the dependent variable. Turning to the selection of control variables, the paper should cite more relevant papers which document the correlations between there control variables with firm innovation. There is a lack of description of 𝛾*­*­­­_t_ and $\mu_{i}$ variables in the model (Are they denotations of year fixed effect and firm fixed effect, respectively?)
4. Methodology: Existing studies have extensively recognized that there are potential endogeneity issues in the regression of firm innovation on subsidies. That means the subsidy is the possible endogenous explanatory variable in the regression (Clausen, 2009 and Li et al., 2021). To address the potential endogeneity issues, I suggest conducting the Difference-in-Difference (DiD) test first, then the authors can run the Difference GMM and System GMM to estimate the model instead of using the static regression as the main methodology. As I perceive that the authors have employed GMM models in robustness checking, I suggest moving this empirical result to the main analyses and citing more supporting literature on the potential endogeneity issue of subsidy variable (the current paper does not cite the relevant studies regarding the issue of endogenous explanatory variable)
5. Analyses of empirical results: The authors do not link the results of the paper to relevant findings in the extant literature. As such, the paper’s results remain isolated and then its contribution is limited.
6. Policy implications: The current content of policy implication section is unlinked to the paper’s findings and somewhat irrelevant to the study. This way, the paper’s results do not provide insights for policy makers. I would prefer to see each policy implication is directly linked to the finding of the paper.

Minor comment

1. The writing is often vague. You need to be as succinct as possible. You should avoid writing such a lengthy and duplicate sentence, as follows “This paper analyzes the mechanism of government subsidies on the innovation of new energy vehicle enterprises and the differences in the impact of different types of enterprises from a quantitative perspective, based on the panel data of new energy vehicle enterprises from 2012-2021, under the micro subject of new energy vehicle enterprises, which enriches the relevant research on new energy vehicle micro enterprises and is of great value to the policy formulation and stimulation of enterprise innovation of new energy vehicles in China. It has important reference value for the policy formulation and stimulation of enterprise innovation in China.”. I suggest dropping the duplicate sentence, breaking down the lengthy sentence to shorter ones. The paper needs to be redrafted and polished thoroughly to improve the conciseness of the paper.
2. The current abstract also needs to be rewritten to remove some duplicates. Again, please avoid using many long-winded sentences (the sentences include many clauses connected by semi-colon) to present the findings in the abstract.
3. Please check for grammatical mistakes and sentence structure as many writing errors appear across the paper. For example, “Differences in property rights, industry chain division of labor, and the number of years an enterprise has been established have different incentives for innovation in government subsidies, which requires "teaching different types of enterprises according to their abilities", giving full play to the advantages of China's state-owned enterprises, while improving the innovative vitality of non-stateowned enterprises; improving the adaptability of the new energy vehicle industry chain division of labor, and setting different incentives for enterprises in different industry chains”. The writing seems to be tedious and needs to be enhanced a lot to meet the standard of an academic research.
4. Please cross-check the reference list and citations to make sure that all citations are listed in the reference.

**Reference**

Clausen, T. H. (2009). Do subsidies have positive impacts on R&D and innovation activities at the firm level?. *Structural change and economic dynamics*, *20*(4), 239-253.

Jiang, C., Zhang, Y., Bu, M., & Liu, W. (2018). The effectiveness of government subsidies on manufacturing innovation: Evidence from the new energy vehicle industry in China. *Sustainability*, *10*(6), 1692.

Li, Q., Wang, M., & Xiangli, L. (2021). Do government subsidies promote new-energy firms’ innovation? Evidence from dynamic and threshold models. *Journal of Cleaner Production*, *286*, 124992.

Shao, W., Yang, K., & Bai, X. (2021). Impact of financial subsidies on the R&D intensity of new energy vehicles: A case study of 88 listed enterprises in China. *Energy Strategy Reviews*, *33*, 100580.
