# Supplementary material for: Government subsidies and innovation in new energy vehicle companies: An empirical study of new energy vehicle listed companies based on Shanghai and Shenzhen A-shares
Source: PLoS One. 2023 Apr 20;18(4):e0284693. doi: 10.1371/journal.pone.0284693 (PMC10118184; doi:10.1371/journal.pone.0284693)
Supplement: Attachment — Submitted filename: Comments to Authors.docx [file pone.0284693.s003.docx]

**Government subsidies and innovation in new energy vehicle companies--Empirical study of new energy vehicle listed companies based on Shanghai and Shenzhen A-shares**

**Comments to Authors:**

The paper is well-written and well-organized. The research method and data analysis are adequately done. However, there are some minor issues that still need to be clarified and strengthened particularly about the theoretical contribution of the research. Please consider the following suggestions:

1. The author must clarify and elaborate more why studying different dimensions of work engagement adds more theoretical contribution to prior study that used aggregate measure of work engagement.
2. Some more recent reference to support the benefits of supervisor support will need to be added.
3. The key assumptions regarding normality need to be explained. It is important to summarize key findings of their study in the abstract. This is important to help readers understand where the paper is going.
4. The policy implications should be strengthened
5. Carefully proofread the article
6. Numbered all equation in the text sequentially.
7. The discussion of the results needs more elaboration. Particularly, more solid discussion how the results advance knowledge in prior research is required.
8. Limitations and Recommendation for Future Research need more elaboration. Actually, limitations and recommendation for future research should be discussed in separated paragraphs.
9. There is no sampling method, how the data was collected was not being revealed. The study also used a single source data, which lead to common method variance, and author do nothing about that a lot more errors in the methodological section.
10. Conclusion should add some latest references to prove the results.

Finally, it’s a nice attempt, the topic is of interest and has a wide range of policy implementations. Bases on the above-mentioned comments, I suggest “**major revision**” of the article for more practicability before “publication”.
