# Supplementary material for: Government subsidies and innovation in new energy vehicle companies: An empirical study of new energy vehicle listed companies based on Shanghai and Shenzhen A-shares
Source: PLoS One. 2023 Apr 20;18(4):e0284693. doi: 10.1371/journal.pone.0284693 (PMC10118184; doi:10.1371/journal.pone.0284693)
Supplement: Attachment — Submitted filename: Response to Reviewers.docx [file pone.0284693.s004.docx]

February 10, 2023

Dear reviewers,

First of all, thank you for your valuable comments, in response to your comments this article has been revised as follows.

Reviewer #1:

1.The current introduction seems to provide too much background information regarding China NEV industry while the motivation and contribution are not made clear. I suggest the following:

Shorten the background information, brief review of the relation between government subsidies and firm innovation in general (i.e., existing evidence of non-linear relationship, crowding-out effect in this relationship),

State the research subject of the paper in the first/ second paragraph of the introduction which is different with the existing studies as it first provides the evidence of inverted U-shape relationship on NEV firm innovation in China.

State the reason why it is important to explore how the effect of government subsidies varies by the role of firms in value chain, the firm type, and the firm age. This would help highlight the main motivation and contribution of the paper in introduction section.

**Response:** The introduction has reduced the description of the background information, highlighted the theme of the study, explained the proposed inverted "U" relationship, added a description of the heterogeneity analysis section and the motivation of the study, and emphasized the contribution of the study.

2.In literature review, the paper provides appropriate hypothesis developments. However, regarding the hypothesis 1, I suggest citing more relevant and recent literature as mentioned above. Please check the duplicate sentences (which happen quite frequently) and redraft the literature review section.

**Response**: The literature review section was extensively revised to provide more arguments for the hypothesis, more recent references were cited, and sentence repetition was thoroughly revised.

3.Regarding variable selection, I perceive that there are many wrong descriptions of the variables used in the model. For example, “Patent¬¬it is the explanatory variable”??? More importantly, the authors did not provide the reasons why they only use Patent as a proxy for firm innovation. Since the major existing study documents that firm innovation is an input-output process which could be proxied by R&D spending and the number of the patents. (Li et al., 2021). On this matter, I would prefer to see more detailed explanation on the selection of the dependent variable. Turning to the selection of control variables, the paper should cite more relevant papers which document the correlations between there control variables with firm innovation. There is a lack of description of 𝛾¬¬¬¬t and μ_i variables in the model (Are they denotations of year fixed effect and firm fixed effect, respectively?)

**Response**: Each of the selected variables is described in detail, and more references are cited as the basis for the selection of variables, and the model explanation is re-stated.

4.Methodology: Existing studies have extensively recognized that there are potential endogeneity issues in the regression of firm innovation on subsidies. That means the subsidy is the possible endogenous explanatory variable in the regression (Clausen, 2009 and Li et al., 2021). To address the potential endogeneity issues, I suggest conducting the Difference-in-Difference (DiD) test first, then the authors can run the Difference GMM and System GMM to estimate the model instead of using the static regression as the main methodology. As I perceive that the authors have employed GMM models in robustness checking, I suggest moving this empirical result to the main analyses and citing more supporting literature on the potential endogeneity issue of subsidy variable (the current paper does not cite the relevant studies regarding the issue of endogenous explanatory variable)

**Response**: For possible endogeneity issues, this paper reinserts two robustness tests, instrumental variation and replacement of explanatory variables, in the robustness test section to mitigate endogeneity. The dynamic panel estimation is moved to the underlying regression. For your proposed DID test, since this paper focuses on the non-linear relationship, it may not be quite applicable to the DID method for the time being, and we did not find any arguments about the DID estimation method for the non-linear relationship, so I hope you will make further pointers. In addition, regional heterogeneity was included in the heterogeneity analysis section, taking into account the regional variability.

5.Analyses of empirical results: The authors do not link the results of the paper to relevant findings in the extant literature. As such, the paper’s results remain isolated and then its contribution is limited.

**Response**: This revision enhances the interpretation of the empirical results and provides more literature support for the validation of the study results.

6.Policy implications: The current content of policy implication section is unlinked to the paper’s findings and somewhat irrelevant to the study. This way, the paper’s results do not provide insights for policy makers. I would prefer to see each policy implication is directly linked to the finding of the paper.

**Response**: Strengthened the correlation between the policy implications section and the study results by making a one-to-one correspondence between the study results and the policy implications.

Reviewer #2

1.The author must clarify and elaborate more why studying different dimensions of work engagement adds more theoretical contribution to prior study that used aggregate measure of work engagement.

**Response**: The innovations and marginal contributions of the study are highlighted in the introduction and literature review section, and more references have been added.

2.Some more recent reference to support the benefits of supervisor support will need to be added.

**Response**: More recent references have been added to highlight the marginal contribution of the study.

3. The key assumptions regarding normality need to be explained. It is important to summarize key findings of their study in the abstract. This is important to help readers understand where the paper is going.

**Response**: The abstract has been reworked to increase the organization of the abstract.

4. The policy implications should be strengthened.

**Response**: Adjustments were made to the policy implications and to correspond to the findings of the study.

5. Carefully proofread the article.

**Response**: Thoroughly revised the article for language errors that appeared in the article.

6. Numbered all equation in the text sequentially

**Response**: The formulas that appear in the article are numbered in order.

7. The discussion of the results needs more elaboration. Particularly, more solid discussion how the results advance knowledge in prior research is required

**Response**: The interpretation of the empirical results is added and correlated with the relevant literature.

8. Limitations and Recommendation for Future Research need more elaboration. Actually, limitations and recommendation for future research should be discussed in separated paragraphs.

**Response**: The conclusion section is reworked. Policy recommendations are re-stated.

9. There is no sampling method, how the data was collected was not being revealed. The study also used a single source data, which lead to common method variance, and author do nothing about that a lot more errors in the methodological section.

**Response**: The sample selection methods, data collection, and empirical methods are explained in more detail, and more references to the literature are provided to support the selection of variables.

10. Conclusion should add some latest references to prove the results

**Response**: More recent literature has been added to verify the reliability of the findings and to correlate with the previous literature.

Thank you again for your valuable suggestions for this revision.

Sincerely

Mingfu Tian
