# Supplementary material for: Government subsidies and innovation in new energy vehicle companies: An empirical study of new energy vehicle listed companies based on Shanghai and Shenzhen A-shares
Source: PLoS One. 2023 Apr 20;18(4):e0284693. doi: 10.1371/journal.pone.0284693 (PMC10118184; doi:10.1371/journal.pone.0284693)
Supplement: Attachment — Submitted filename: Review Report_PONE-D-22-30783R1.docx [file pone.0284693.s005.docx]

Review report on the manuscript PONE-D-22-30783R1

In the revised manuscript, the authors have seriously addressed the suggested comments.

Thank you for your revision.

I just have several minor notes on the writing of the paper as follows:

1. You should present the findings of the paper in present tense, not in past tense as in the current revised version.
2. If possible, you should use professional editing and proof reading to enhance the writing of the paper. ***Too many writing errors need to be corrected***. For example, “First, government subsidies have incentive effect on enterprise innovation[8] , second, government subsidies inhibit enterprise innovation[9], and third, there is no significant effect[10], most of these literatures only consider the linear relationship between government subsidies and enterprise innovation, and less literature has more in-depth research on nonlinear mechanism.” or “The possible reason is that because SOEs will have the underwriting guarantee from the government, they may form a dependence effect on innovation incentives due to this underwriting guarantee and will not be eliminated from the market due to the decline of innovation ability, while non-SOEs must continuously innovate their products to adapt to the severe market competition due to their self-sustaining characteristics, and the characteristics of market economy determine the inevitability of innovation incentives for non-SOEs.” Such the lengthy sentences need to be redrafted. ***Not limited in the given examples, please check the whole paper for the same errors.***
3. Please note that the selling point of your paper is that it first provides the evidence of inverted U-shape relationship between government subsidies and firm innovation in NEV industry in China. Meanwhile, ***you didn’t mention this main contribution in the current introduction***. Do not state that “less literature has more in-depth research on nonlinear mechanism” and thereby your paper fills this void in the literature. As the reviewed literature shows that there are abundant studies exploring the nonlinear relationship between subsidies and firm innovation in China (see the references in my last review report). Highlight the selling point of your study in the introduction.
4. My suggested DID model can be used together with your instrumental variable analysis to test the causal effect of subsidy on firm innovation with the aim of reducing the endogeneity concerns as a robustness check. However, as your study has conducted several robustness checks, I perceive that the paper provides sufficient evidence to state the consistency of the results. Even though no further robustness check is required in this paper, you can refer to the research of Black and Kim (2012) to see how several methods are combined to address the endogeneity concern in panel regression (For your interest only)
