# Supplementary material for: Government subsidies and innovation in new energy vehicle companies: An empirical study of new energy vehicle listed companies based on Shanghai and Shenzhen A-shares
Source: PLoS One. 2023 Apr 20;18(4):e0284693. doi: 10.1371/journal.pone.0284693 (PMC10118184; doi:10.1371/journal.pone.0284693)
Supplement: Attachment — Submitted filename: PLOSE ONE RP-1.docx [file pone.0284693.s006.docx]

**Government subsidies and innovation in new energy vehicle companies: Empirical study of new energy vehicle listed companies based on Shanghai and Shenzhen A-shares**

**Comments for author:**

The subject is interesting and has a wide variety of policy implications, I suggest “acceptance” for the article.
